# Supplementary material for: Exploring positive experiences of primary and secondary caregivers of older persons in resource-limited urban settings in Accra, Ghana
Source: PLoS One. 2022 Apr 1;17(4):e0266269. doi: 10.1371/journal.pone.0266269 (PMC8975136; doi:10.1371/journal.pone.0266269)
Supplement: S1 File — (DOCX) [file pone.0266269.s001.docx]

**Interview Guide for Qualitative Study**

**Thank you for agreeing to participate in this research on family caregiving to the elderly.**

1. Please, can you tell me a little about yourself?

**Probe:** Life before being a caregiver, social dynamics (social activities, friends, etc.)

1. What does it mean to provide care?

**Motivation for providing care**

1. How did you become a caregiver to the elderly?

**Probe:** What happened before you became a caregiver?

Who decided that you should become a caregiver?

Did you like the idea of becoming a caregiver? **Probe:** Why?

1. What are the reasons why you provide care to the elderly?

**Probe:** Obligatory/no choice, Reciprocity, Benefit from someone else not care receiver etc.

1. Who do you think should provide care for the elderly? **Probe:** Why?
2. What are the cultural values or beliefs associated with caregiving for the elderly in this community?

**Caregiving experiences**

1. Have you ever provided care to a friend or family member who needed care in the past?

**Probe:** If yes, who was this person?

1. Are you the main person providing care to the elderly?

**Probe:** Do you do it all the time?

Do other family members or friends assist you in caring for the elderly?

1. How long have you been providing care to the elderly (years and/or months)?
2. How many hours per day on average do you usually spend providing care for the elderly?
3. What types of care/support do you provide to the elderly?

**Probe:** Eating; bathing; dressing; paying for food, bills, medicine; administering medicines, emotional support such as company, etc.

1. How do you feel about providing personal care to the elderly?

**Probe:** How do you feel about providing bathing to the elderly? **Why?**

How do you feel about providing dressing to the elderly? **Why?**

How do you feel about providing toileting (getting to and using the toilet) to the elderly? **Why?**

1. Kindly narrate what you do for your elderly care recipient on a typical day?
2. If you could draw a picture of you doing caregiving, what would it look like?
3. What has the experience been like providing care to the elderly? **Why?**
4. How has caregiving impacted on your everyday life?
5. Some caregivers are of the view that caregiving is beneficial to them. What do you think about it?

**Probe:** What makes it beneficial?

1. Have you benefited from caregiving to the elderly? **Kindly narrate the occasion**

**Coping strategies**

1. How do you cope with these challenges as an elderly caregiver?
2. In relation to providing care and support to the elderly, have you received any support, help or assistance?

**If yes,** who provided this support, help or assistance? **Probe:** What was the reason(s) for providing the support, help or assistance?

1. What was the type of support, help or assistance you received?

**Probe:** Financial, emotional, health, physical, personal

**Perception and other issues**

1. In your community, what are people’s perceptions of caregiving to the elderly?
2. What more do you wish you had known about while caring for the elderly?

**Probe:** Do you know where you can get this information?

1. If you were advising a relative/friend about caregiving, what advise would you give them?
2. Is there any other issue related to your caregiving experience that you feel is important that we have not brought up in this interview?

**This is the end of the interview. Thank you for your time!**
