# Supplementary material for: Exploring positive experiences of primary and secondary caregivers of older persons in resource-limited urban settings in Accra, Ghana
Source: PLoS One. 2022 Apr 1;17(4):e0266269. doi: 10.1371/journal.pone.0266269 (PMC8975136; doi:10.1371/journal.pone.0266269)
Supplement: S1 Appendix — (DOCX) [file pone.0266269.s002.docx]

**Themes and their sample quotes for rewards family caregiver derived from providing care to their care recipients**

| **Themes** | **Categories** | **Subcategories** | **Sample of significant statements** |
| --- | --- | --- | --- |
| Tangible rewards derived from providing care to their care recipients | Gifts received from care recipients, relatives, friends, and church | Cash gifts received from care recipients, relatives and church | *“Due to the care I provide to my mum, the church we attend sometimes gives me money to motivate me to continue to provide care to her.”* (R11)  *“There are times she [care recipient] helps me pay my children school fees.”* (R27)  *“Sometimes when I am ‘broke’, my mum supports me financially.”* (R31) |
|  |  | In-kind gifts received from care recipients, relatives, and friends | *“I sometimes get some provisions due to the care I provide for my mum.”* (R11)  *“She [care recipient] gives me rice, oil, biscuit and exercise books for my children.”* (R27)  *“I come [to the care recipient] for milk, provisions, bread, and other supplies. When she has food, she gives me.”* (R29)  *“I have received some gifts from friends and family members due to the care I provide for my mum.”* (R31) |
|  |  | Real estate received from care recipient | *“I have a room [in our family house] and it is because of the care I provide to her.”* (R9)  *“He [care recipient] has given me a piece of land in Kokrobite to build a house. He has been pressuring me to build on it but the challenge is money.”* (R18) |
| Intangible rewards derived from providing care to their care recipients | Blessings from God due to providing care |  | *“All the time, I am being delivered. Like the other time, a tragedy occurred, a serious one. The shop that you see over there [participant pointed to a shop], I hope you have seen the violet shop? We just rebuilt it. It collapsed and I was inside it. Two vehicles crushed and entered it. It even hit me but by the Grace of God, nothing happened to me. So you see, the good things that I do, the good that I use to do to people, you see that the blessing is following me”.* (R4)  *“Since I started providing care for my mum, I have never lacked anything. The Good Lord blesses me and sees to it that we never lack anything that we need.”* (R5) |
|  | Skills acquisition from providing care | Skills in providing care to older persons | *“It has helped me to know how to take care of older persons because at first, I didn’t know how to do it.”* (R12) |
|  |  | Learnt to perform household chores | *“It has taught me how to cook and do household chores.”* (R3) |
|  | Enhanced personal attributes of family caregiver | Appreciation of caregiving efforts | *“People even tell me that I have done well by taking care good care of my mum. During the Easter celebration, we went to church and when people saw her, they gave me a handshake that I have done well by taking good care of my mum. When they do that, I feel appreciated.”* (R5) |
|  |  | Feeling valued due to privileges caregiving has bestowed on family caregiver | *“… I feel I am important because I get to sit at places where I would not sit if I was alone. I get to sit at the high table when I accompany her to family meetings. If it was not because of her, I will not be sitting there because my age doesn’t qualify me.”* (R6)  *“Because of the care I provide to my care recipient, I can attend family meetings. If not for her, my age would have disqualified me from attending such meetings.”* (R24) |
|  |  | Learnt humility and patience due to providing care | *“It has taught me to be humble and patient in whatever I do.”* (R17) |
|  |  | Learnt to be compassionate due to providing care | *“The help I give to him has helped me learn a lot of things. Some of the things that I have learned are that if someone is in need and you are capable you should help him or her.”* (R2) |
|  | Family cohesion among family due to providing care |  | *“There used to be some quarrels in the family but since I started providing care to my mum it has brought the family together.”* (R5) |
|  | Health consciousness of conditions affecting care recipient |  | *“Health-wise, I have learned a lot of things that affect the family that can equally affect me so I am taking precautions now and then to make sure I don’t fall a victim. So I check my diet.”* (R26) |
