# Supplementary material for: Exploring positive experiences of primary and secondary caregivers of older persons in resource-limited urban settings in Accra, Ghana
Source: PLoS One. 2022 Apr 1;17(4):e0266269. doi: 10.1371/journal.pone.0266269 (PMC8975136; doi:10.1371/journal.pone.0266269)
Supplement: Attachment — Submitted filename: EXPLORING POSITIVE EXPERIENCES-2.docx [file pone.0266269.s003.docx]

**PLOS ONE**

### Exploring positive experiences of primary and secondary caregivers of older persons in resource-poor urban settings in Accra, Ghana

--Manuscript Draft--

| **Manuscript Number:** | PONE-D-21-29801 |
| --- | --- |
| **Article Type:** | Research Article |
| **Full Title:** | Exploring positive experiences of primary and secondary caregivers of older persons in resource-poor urban settings in Accra, Ghana |
| **Short Title:** | Exploring positive experiences of primary and secondary caregivers of older persons |
| **Corresponding Author:** | Frank Kyei-Arthur, Ph.D.  University of Environment and Sustainable Development Somanya, Eastern Region GHANA |
| **Keywords:** | positive experiences; primary caregivers; secondary caregivers; resource-poor urban settings; Accra; Ghana |
| **Abstract:** | Family caregivers experience both negative and positive outcomes. However, most studies have mainly focused on the negative outcomes. In addition, few studies have focused on both primary and secondary caregivers. This study explored the positive experiences of both primary and secondary caregivers of older persons in resource- poor urban settings in Accra, Ghana. Thirty-one family caregivers were interviewed in James Town and Ussher Town. The interviews were audio-recorded, transcribed verbatim, and analysed thematically using NVivo 10. The findings show that the primary and secondary caregivers derived tangible and intangible rewards from providing care to older persons. The tangible rewards included gifts while the intangible include blessings, skills acquisition, enhanced personal attributes, family cohesion, and health consciousness. Future studies need to longitudinally examine the positive experiences of primary and secondary caregivers to provide research evidence to policymakers so that they can understand the complexities of caregiving and thereby implement appropriate interventions. |
| **Order of Authors:** | Frank Kyei-Arthur, Ph.D. |
|  | Samuel Nii Ardey Codjoe, Ph.D. |
|  | Delali Margaret Badasu |
| **Additional Information:** |  |
| **Question** | **Response** |
| **Financial Disclosure**  Enter a financial disclosure statement that describes the sources of funding for the work included in this submission. Review the [submission guidelines](http://journals.plos.org/plosone/s/submission-guidelines#loc-financial-disclosure-statement) for detailed requirements. View published research articles from [*PLOS ONE*](http://journals.plos.org/plosone/) for specific examples.  This statement is required for submission and **will appear in the published article** if the submission is accepted. Please make sure it is accurate. | The author(s) received no specific funding for this work. |

| **Unfunded studies**  Enter: *The author(s) received no specific funding for this work.*  **Funded studies**  Enter a statement with the following details:   - Initials of the authors who received each award - Grant numbers awarded to each author - The full name of each funder - URL of each funder website - Did the sponsors or funders play any role in the study design, data collection and analysis, decision to publish, or preparation of the manuscript? - **NO** - Include this sentence at the end of your statement: *The funders had no role in study design, data collection and analysis, decision to publish, or preparation of the manuscript.* - **YES** - Specify the role(s) played. |  |
| --- | --- |
| * typeset |  |
| **Competing Interests**  Use the instructions below to enter a competing interest statement for this submission. On behalf of all authors, disclose any [competing interests](http://journals.plos.org/plosone/s/competing-interests) that could be perceived to bias this work—acknowledging all financial support and any other relevant financial or non- financial competing interests.  This statement is required for submission and **will appear in the published article** if the submission is accepted. Please make sure it is accurate and that any funding sources listed in your Funding Information later in the submission form are also declared in your Financial Disclosure statement.  View published research articles from  [*PLOS ONE*](http://journals.plos.org/plosone/) for specific examples. | The authors have declared that no competing interests exist. |

| **NO authors have competing interests**  Enter: *The authors have declared that no competing interests exist.*  **Authors with competing interests**  Enter competing interest details beginning with this statement:  *I have read the journal's policy and the authors of this manuscript have the following competing interests: [insert competing interests here]* |  |
| --- | --- |
| * typeset |  |
| **Ethics Statement**  Enter an ethics statement for this submission. This statement is required if the study involved:   - Human participants - Human specimens or tissue - Vertebrate animals or cephalopods - Vertebrate embryos or tissues - Field research   Write "N/A" if the submission does not require an ethics statement.  General guidance is provided below. Consult the [submission guidelines](http://journals.plos.org/plosone/s/submission-guidelines#loc-guidelines-for-specific-study-types) for detailed instructions. **Make sure that all information entered here is included in the Methods section of the manuscript.** | The study was approved by the Ethics Committee for the Humanities (ECH) of the University of Ghana (ECH 009/16-17). Written consent was obtained from participants. |

| **Format for specific study types**  **Human Subject Research (involving human participants and/or tissue)**   - Give the name of the institutional review board or ethics committee that approved the study - Include the approval number and/or a statement indicating approval of this research - Indicate the form of consent obtained (written/oral) or the reason that consent was not obtained (e.g. the data were analyzed anonymously)   **Animal Research (involving vertebrate animals, embryos or tissues)**   - Provide the name of the Institutional Animal Care and Use Committee (IACUC) or other relevant ethics board that reviewed the study protocol, and indicate whether they approved this research or granted a formal waiver of ethical approval - Include an approval number if one was obtained - If the study involved *non-human primates*, add *additional details* about animal welfare and steps taken to ameliorate suffering - If anesthesia, euthanasia, or any kind of animal sacrifice is part of the study, include briefly which substances and/or methods were applied   **Field Research**  Include the following details if this study involves the collection of plant, animal, or other materials from a natural setting:   - Field permit number - Name of the institution or relevant body that granted permission |  |
| --- | --- |
|  |  |
| **Data Availability**  Authors are required to make all data underlying the findings described fully available, without restriction, and from the time of publication. PLOS allows rare exceptions to address legal and ethical concerns. See the [PLOS Data Policy](http://journals.plos.org/plosone/s/data-availability) and [FAQ](http://journals.plos.org/plosone/s/data-availability#loc-faqs-for-data-policy) for detailed information. | Yes - all data are fully available without restriction |

| A Data Availability Statement describing where the data can be found is required at submission. Your answers to this question constitute the Data Availability Statement and **will be published in the article**, if accepted. |  |
| --- | --- |
| **Important:** Stating ‘data available on request from the author’ is not sufficient. If your data are only available upon request, select ‘No’ for the first question and explain your exceptional situation in the text box. |  |
| Do the authors confirm that all data underlying the findings described in their manuscript are fully available without restriction? |  |
| **Describe where the data may be found in full sentences. If you are copying our sample text, replace any instances of XXX with the appropriate details.**   - If the data are **held or will be held in a public repository**, include URLs, accession numbers or DOIs. If this information will only be available after acceptance, indicate this by ticking the box below. For example: *All XXX files are available from the XXX database (accession number(s) XXX, XXX.*). - If the data are all contained **within the manuscript and/or Supporting Information files**, enter the following: *All relevant data are within the manuscript and its Supporting Information files.* - If neither of these applies but you are able to provide **details of access elsewhere**, with or without limitations, please do so. For example:   *Data cannot be shared publicly because of [XXX]. Data are available from the XXX Institutional Data Access / Ethics Committee (contact via XXX) for researchers who meet the criteria for access to confidential data.*  *The data underlying the results presented in the study are available from (include the name of the third party* | All relevant data are within the manuscript and its Supporting Information files. |

| *and contact information or URL).*   - This text is appropriate if the data are owned by a third party and authors do not have permission to share the data.   * typeset |  |
| --- | --- |
| Additional data availability information: |  |

Manuscript	[Click here to](https://www.editorialmanager.com/pone/download.aspx?id=29841031&guid=58441446-03c4-408c-ab9c-72c4b6fef969&scheme=1)

[access/download;Manuscript;Manuscript_17Sept21.docx](https://www.editorialmanager.com/pone/download.aspx?id=29841031&guid=58441446-03c4-408c-ab9c-72c4b6fef969&scheme=1)

1

2

3

##### 4 Exploring positive experiences of primary and secondary caregivers of older persons in

5 **resource-poor urban settings in Accra, Ghana**

6

7

8	Frank Kyei-Arthur^1¶^*, Samuel Nii Ardey Codjoe ^2&^, Delali Margaret Badasu ^2&^

9

10

11 ^1^ Department of Environment and Public Health, University of Environment and Sustainable
12 Development, Somanya, Eastern Region, Ghana
13 ^2^ Regional Institute for Population Studies, University of Ghana, Legon, Accra, Ghana

14

15

16 * Corresponding author
17 E-mail: [fkyeiarthur@yahoo.com](mailto:fkyeiarthur@yahoo.com) (FK-A)

18

19

20 ^¶^ These authors contributed equally to this work.
21 ^&^ These authors contributed equally to this work.

22

23

24

# 25 Abstract

26 Family caregivers experience both negative and positive outcomes. However, most studies have
27 mainly focused on the negative outcomes. In addition, few studies have focused on both primary
28 and secondary caregivers. This study explored the positive experiences of both primary and
29 secondary caregivers of older persons in resource-poor urban settings in Accra, Ghana. Thirty-
30 one family caregivers were interviewed in James Town and Ussher Town. The interviews were
31 audio-recorded, transcribed verbatim, and analysed thematically using NVivo 10. The findings
32 show that the primary and secondary caregivers derived tangible and intangible rewards from
33 providing care to older persons. The tangible rewards included gifts while the intangible include
34 blessings, skills acquisition, enhanced personal attributes, family cohesion, and health
35 consciousness. Future studies need to longitudinally examine the positive experiences of primary
36 and secondary caregivers to provide research evidence to policymakers so that they can
37 understand the complexities of caregiving and thereby implement appropriate interventions.
38 **Keywords:** positive experiences, primary caregivers, secondary caregivers, resource-poor urban
39 settings, Accra, Ghana

40

41

42

43

44

45

# 46 Introduction

47 Globally, there has been an increase in the proportion of older persons (aged 65 years and older).
48 Between 1990 and 2021, the proportion of the population of older people to total global population increased from 6 percent
49 to 10 percent, and it is estimated to reach 16 percent in 2050 [1, 2]. In Ghana, the proportion
50 has increased from 3 percent in 1960 to 4 percent in 2021 [2, 3]. By 2050, it is estimated that
51 about 9 percent of Ghanaians will be aged 65 years and older. The increase in the proportion of
52 older persons can be attributed to increasing longevity and declining fertility [4].
53 Older persons, especially those aged 75 years and over, may need some level of assistance due to
54 their functional limitations. In Ghana and other sub-Saharan African countries, the family is the
55 main provider of care and support to older persons [3, 5, 6]. The provision of unpaid care to a
56 relative, partner, friend, or neighbour who needs care is referred to as family caregiving [7].
57 Family caregiving roles are often performed by women [8-10]. Women often provide personal
58 care (such as bathing, cooking, laundry, and dressing) while men provide financial care (such as
59 providing money for food and paying medical and other types of bills) [9, 11].
60 In Ghana, modernisation processes (such as urbanisation) have influenced the role of the family
61 in providing care to older persons due to financial constraints and increased involvement of
62 women in the paid labour market [12-14]. Also, poverty in coastal urban areas (including the
63 study area, Ga Mashie) has worsened over time [15] and this has repercussions for financial care
64 to older persons.
65 Previous studies have established that family caregivers experience both negative and positive
66 outcomes from performing their roles and responsibilities [16-19]. However, most studies on
67 family caregiving to older persons have mainly focused on the negative outcomes to the neglect
68 of the positive outcomes [19-21]. In this regard, previous studies have established that family
69 caregivers of older persons experience financial constraints, poor physical and mental health, and

70	weight loss [9, 13, 22, 23].

71 In addition, family caregivers often share their roles and responsibilities with others [24, 25]. In
72 every caregiving situation, there is always someone who provides the most care. This person is
73 referred to as primary caregiver while others who support this main person are known as
74 secondary caregivers. However, most studies on family caregiving to older persons have mainly
75 focused on primary caregivers [26-30] with limited studies on both primary and secondary
76 caregivers [13, 23, 31]. The main focus on primary caregivers is partly because they provide
77 most care and may experience a greater brunt of the negative outcomes of family caregiving.
78 Nevertheless, family caregiving is dynamic and caregiving roles change over time [19].
79 The available literature does not indicate that any study has explored the positive experiences of
80 both primary and secondary caregivers of older persons in Ghana and sub-Saharan Africa in
81 general. The paucity of studies on the positive experiences of primary and secondary caregivers
82 in Ghana and sub-Saharan Africa limits researchers' and policymakers’ understanding of the
83 nuances of family caregiving experiences which would enable them to design appropriate
84 interventions to address the associated challenges. Accordingly, this study explored the positive
85 experiences of primary and secondary caregivers in urban poor communities in Accra, the capital
86 city of Ghana. It specifically explored the rewards that primary and secondary caregivers derived
87 from providing care to older persons.

88

89

## 90 Conceptual framework

91 This study was guided by Kramer’s model of caregiver adaptation [32]. She highlights that well-
92 being outcomes (whether positive or negative) are influenced by the background and context of
93 caregiving, and intervening processes. According to Kramer [32], the background and context of
94 caregiving component consist of caregiver characteristics (namely age, gender, duration of
95 caregiving activities, and employment status), care recipient characteristics (that is the type of
96 illness, the severity of illness, care recipient needs), and caregiver attitudinal variables and efforts
97 (such as motivations for providing care). Resources (such as social support and coping
98 strategies), and appraisal of role gain and strain in the form of caregiving satisfaction and burden
99 were identified as factors that enhance, and reduce positive and negative outcomes of caregivers
100 respectively.
101 Although Kramer’s model of caregiver adaptation discusses both positive and negative
102 outcomes, this study focused on the positive outcomes of caregivers. Therefore, it adopted and
103 modified Kramer’s model of caregiver adaptation (Fig 1). Positive outcomes were
104 conceptualised as the rewards caregivers derive from providing care to their care recipients.
105 This manuscript is derived from a broader thesis which collected data on characteristics of family
106 caregivers and their care recipients, and caregivers motivations for providing care to their care
107 recipients [33]. In addition, data were collected on family caregivers’ social support and coping
108 strategies. Previous studies have documented that background and context of caregiving, and
109 intervening processes influence caregiver well-being outcomes [34-36]. We also conceptualised
110 that the background and context of caregiving can directly influence positive outcomes.
111 This manuscript does not seek to assess the pathways to positive caregiving, but it seeks to describe
112 positive outcomes among family caregivers in a context that has rarely been studied.
113 113

##### 114 Fig 1. Conceptual framework for positive experiences of primary and secondary caregivers

115 115

# 116 Methods

## 117 Study setting

118 This study was conducted in James Town and Ussher Town, which are neighbouring towns in
119 the city of Accra, commonly referred to as Ga Mashie. Ga Mashie is one of the six indigenous
120 Ga localities and one of the oldest localities in Accra, the capital city of Ghana [37]. They are
121 located on the Gulf of Guinea and recognised as resource-poor urban localities in Accra [38].
122 They are densely populated and residents lack access to basic social amenities such as potable
123 water and waste disposal. Fishing and fishing-related activities are the main economic activities
124 of residents. Residents in James Town and Ussher Town dwell in multigenerational households.
125 Type of study:

## 126 Data collection

127 The data collected for this study is from the third wave of the Regional Institute for Population
128 Studies’ (RIPS) Urban Health and Poverty Survey (UPHS) which took place in November 2016.
129 The UPHS is a longitudinal survey that examines the health, poverty, and development nexus in
130 Agbogbloshie, James Town, and Ussher Town. The third wave of the UPHS was funded by
131 RIPS. Family caregivers were purposely selected from a sample of family caregivers who
132 indicated in the third wave of the UPHS that they or a member of their household provided care
133 to care recipients because they were old and weak. The third wave of the UPHS had 57 family
134 caregivers. The first author and research assistants were able to track 30 out of the 57 family
135 caregivers. Three out of the 30 family caregivers identified were excluded from the study since
136 their care recipients had died more than 6 months preceding the data collection.
137 During the data collection, care recipients of all identified family caregivers were interviewed
138 first to identify all family caregivers who assisted them since the study was interested in both
139 primary and secondary caregivers. Family caregivers who met the following criteria were
140 interviewed: they must be 18 years and over; a relative or non-relative, or friend of the care
141 recipient; currently providing the unpaid care to an older person; and should have provided the
142 unpaid care for at least six months prior to the data collection.
143 In total, twenty in-depth interviews were conducted with care recipients while thirty-one family
144 caregivers were interviewed. The in-depth interviews were conducted in the *Ga* language by
145 three trained field assistants. The interview guide covered topics on motivation for providing
146 care, caregiving experiences, coping strategies, perception and other issues (See Supplementary
147 File 1). The interviews ranged between 30 and 55 minutes. Ethical clearance for the study was
148 approved by the Ethics Committee for the Humanities (ECH) of the University of Ghana (ECH
149 009/16-17). Participation in the study was voluntary and informed consent of caregivers was
150 sought before they were interviewed.

## 151 Data analysis

152 All the interviews were audio-taped and transcribed verbatim from the *Ga* language into English.
153 The interview transcripts were quality checked and analysed using the thematic analysis
154 approach [39] with the aid of NVivo 10 software. All the transcripts were read and emerging
155 codes were identified and grouped into themes. Deductive codes were developed from previous
156 studies on family caregiving to older persons [18, 40, 41], while inductive codes were developed
157 as they emerged from the transcripts of family caregivers.
158 All the transcripts were read several times to ensure comprehensive understanding of the data. Codes were then assigned to important text in the form of categories and subcategories using NVivo. These categories and subcategories were then aggregated to develop themes of positive experiences of caregiving.
159 Trustworthiness of findings is essential in qualitative research. To achieve trustworthiness in
160 qualitative studies, numerous strategies have been recommended including thick description,
161 peer review/debriefing, development of a coding system, and member checking [42]. To achieve
162 trustworthiness in the study, the researchers worked collaboratively and closely and consulted peers
163 during the entire data analysis process to ensure that data were analysed from the perspectives of
164 family caregivers. Their feedbacks on quotes and/or themes were addressed by re-analysing/re-
165 examining those quotes and/or themes. Also, the findings of the study after data analysis were
166 shared with family caregivers to confirm if the analysis reflected their experiences or provide
167 additional information. Eight family caregivers accepted to go through the analysis and
168 confirmed the analysis reflected their experiences. They, however, did not provide any additional
169 information.
170 172

# 171 Results

## 172 Socio-demographic characteristics of the family caregivers

173 Table 1 presents the socio-demographic characteristics of family caregivers. Family caregivers
174 were aged between 21 and 76 years. Out of the 31 family caregivers, 15 were primary caregivers
175 while the remaining 16 were secondary caregivers. Most primary and secondary caregivers were
176 females (n = 26), and Ga-Dangme (n = 24). The majority of the primary caregivers (n = 15) had
177 formal education, while all 16 secondary caregivers had formal education. Most primary and
178 secondary caregivers (n = 24) were employed and more than half (n = 18) worked at home or
179 close to home. In addition, 27 of the respondents
180 were Christians and 20 had ever married.
181 Furthermore, majority of the caregivers (n = 22) lived in James Town. Nine of the primary caregivers and eight (8) of the secondary caregivers were sons/daughters relatives of the care recipients respectively.

##### 182 Table 1. Characteristics of family caregivers

|  | **Primary caregivers (n = 15)** | | **Secondary caregivers (n = 16)** | |
| --- | --- | --- | --- | --- |
|  | **Frequency** | **Percent** | **Frequency** | **Percent** |
| **Age** |  |  |  |  |
| Range | 25 - 76 |  | 21 - 70 |  |
| Mean (years) | 44.9 |  | 45.9 |  |
| **Sex** |  |  |  |  |
| Male | 4 | 26.7 | 1 | 6.3 |
| Female | 11 | 73.3 | 15 | 93.7 |
| **Ethnicity** |  |  |  |  |
| Ga-Dangme | 13 | 86.7 | 11 | 68.7 |
| Akan | 2 | 13.3 | 2 | 12.5 |
| Other | 0 | 0.0 | 3 | 18.8 |
| **Education** |  |  |  |  |
| No education | 1 | 6.7 | 0 | 0.0 |
| Primary | 5 | 33.3 | 2 | 12.5 |
| Junior High School/Middle | 3 | 20.0 | 11 | 68.7 |
| Secondary/Senior High School | 3 | 20.0 | 2 | 12.5 |
| Higher | 3 | 20.0 | 1 | 6.3 |
| **Employment** |  |  |  |  |
| Unemployed | 3 | 20.0 | 4 | 25.0 |
| Full-time | 10 | 66.7 | 8 | 50.0 |
| Part-time | 2 | 13.3 | 4 | 25.0 |
| **Place of Work** | **(n = 12)** |  | **(n = 12)** |  |
| At home | 2 | 16.7 | 4 | 33.3 |

| Away from home, nearby | 6 | 50.0 | 6 | 50.0 |
| --- | --- | --- | --- | --- |
| Away from home, far away | 4 | 33.3 | 2 | 16.7 |
| **Religion** |  |  |  |  |
| Christian | 14 | 93.3 | 13 | 81.2 |
| Muslim | 1 | 6.7 | 3 | 18.8 |
| **Marital Status** |  |  |  |  |
| Never married | 4 | 26.7 | 7 | 43.8 |
| Currently married | 7 | 46.6 | 2 | 12.4 |
| Formerly married | 4 | 26.7 | 7 | 43.8 |
| **Locality** |  |  |  |  |
| James Town | 11 | 73.3 | 11 | 68.7 |
| Ussher Town | 4 | 26.7 | 5 | 31.3 |
| **Relationship with care recipient** |  |  |  |  |
| Spouse | 1 | 6.7 | 0 | 0.0 |
| Son/daughter | 9 | 60.0 | 6 | 37.5 |
| Other relative | 5 | 33.3 | 8 | 50.0 |
| Friend | 0 | 0.0 | 2 | 12.5 |

183 Source: Informal Caregiving Study, 2016

## 184 Positive experiences of the primary and secondary caregivers

185 Several types of rewards were received by the primary and secondary caregivers and they are
186 described in the succeeding sections. Primary and secondary caregivers received tangible and
187 intangible rewards. Table 2 shows the themes, categories and subcategories.
188 193
189 **Table 2. Themes, categories and subcategories**

| **Themes** | **Categories** | **Sub-categories** |
| --- | --- | --- |
| Tangible rewards | Gifts | Cash |
|  |  | In-kind |
|  |  | Real estate |
| Intangible rewards | Blessings |  |
|  | Skills acquisition | Skills in providing care |
|  |  | Performing household chores |
|  |  | Communicating with older persons |
|  | Enhanced personal attributes | Appreciation |
|  |  | Feeling valued |
|  |  | Humility and patience |
|  |  | Compassion |
|  | Family cohesion |  |
|  | Health consciousness |  |

190 **Tangible rewards**
191 **Gifts.** Gifts emerged as a tangible reward for caregiving to older persons. A total of 21 primary
192 and secondary caregivers received gifts as rewards for the provision of care to their care
193 recipients. They received three types of gifts namely: (1) cash; (2) in-kind; and (3) real estate.
194 Care recipients sometimes financially supported their caregivers when they were financially
195 constrained. Some of them assisted their caregivers to pay their children’s school fees. Relatives
196 of the care recipients and churches of both care recipients and caregivers also provided cash to
197 family caregivers to motivate them to continue their care provision. A total of 16 caregivers who
198 received financial gifts were females. The ensuing quotes highlight these financial gifts
199 caregivers received.
200 *“Due to the care I provide to my mum, the church we attend sometimes gives me money*
201 *to motivate me to continue to provide care to her.”* (Female, 65 years, Primary caregiver,
202 James Town)
203 *“Sometimes when I am ‘broke’, my mum supports me financially.”* (Female, 45 years,
204 Primary caregiver, Ussher Town)
205 In addition, the care recipients, relatives, and friends gave other tangible gifts including bread,
206 biscuits, cooking oil, and rice to family caregivers. Family caregivers who received in-kind gifts
207 were females. Some primary and secondary caregivers explained that:
208 *“I come [to the care recipient] for milk, provisions, bread, and other supplies. When she*
209 *has food, she gives me.”* (Female, 22 years, Secondary caregiver, Ussher town)
210 *“I sometimes get some provisions due to the care I provide for my mum.”* (Female, 65
211 years, Primary caregiver, James Town)
212 Family caregivers also received land and free room/accommodation as a reward for caregiving.
213 One primary caregiver mentioned that he received a piece of land from his father in appreciation
214 for his caregiving roles and responsibilities. He narrated that:
215 *“He [care recipient] has given me a piece of land in Kokrobite to build a house. He has*
216 *been pressuring me to build on it but the challenge is money.”* (Male, 28 years, Primary
217 caregiver, James Town)
218 In addition, a male primary caregiver noted that the care he provided to his grandmother enabled
219 him to get free room/accommodation in their family house which would have been impossible
220 due to his young age. He explained that:

| 226 | *“I have a room [in our family house] and it is because of the care I provide to her.”* |
| --- | --- |
| 227 | (Male, 25 years, Primary caregiver, James Town) |
| 228 |  |

### 229 Intangible rewards

230 Five emerging themes from intangible rewards were stated: (1) blessings; (2) skills acquisition;
231 (3) enhanced personal attributes; (4) family cohesion; and (5) health consciousness.
232 **Blessings.** Seven caregivers (2 males and 5 females) stated that they received blessings from
233 God due to the care they provide to their care recipients and it has consequently opened up
234 opportunities for them. A female primary caregiver explained that:
235 *“Since I started providing care for my mum, I have never lacked anything. The Good*
236 *Lord blesses me and sees to it that we never lack anything that we need.”* (Female, 40
237 years, Primary caregiver, James Town)
238 The blessings received from their caregiving manifested in various forms. A secondary caregiver
239 explained that he was saved from an accident due to the financial care he provided his care
240 recipients. He narrated the following:
241 *“All the time, I am being delivered. Like the other time, a tragedy occurred, a serious*
242 *one. The shop that you see over there [participant pointed to a shop], I hope you have*
243 *seen the violet shop? We just rebuilt it. It collapsed and I was inside it. Two vehicles*
244 *crushed and entered it. It even hit me but by the Grace of God, nothing happened to me.*
245 *So you see, the good things that I do, the good that I use to do to people, you see that the*
246 *blessing is following me”.* (Male, 21 years, Secondary caregiver, James Town)
247 247
248 **Skills acquisition.** The primary and secondary caregivers described their skill acquisition in
249 three areas: (1) skills in providing care; (2) performing household chores; and (3) communicating
250 with older persons.
251 **Skills in providing care.** Four caregivers who provided physical care (such as dressing up the
252 care recipient, fetching water for bathing, cooking, and cleaning care recipients when they soil
253 themselves) reported that they had acquired skills in caring for older persons. These caregivers
254 said that they had no prior experience in providing care to older persons before they took on their
255 current caregiving roles and responsibilities. All the caregivers who acquired skills in caring for
256 older persons were females. A female primary caregiver who cooks and dresses her grandmother
257 stated the following:
258 *“It has helped me to know how to take care of older persons because at first, I didn’t*
259 *know how to do it.”* (Female, 36 years, Primary caregiver, James Town)
260 **Performing household chores.** Two family caregivers (1 primary and 1 secondary) narrated that
261 the provision of care to their care recipients has helped them to learn how to perform household
262 chores such as cooking. A secondary caregiver said:
263 *“It has taught me how to cook and do household chores.”* (Female, 30 years, Secondary
264 caregiver, James Town)
265 **Communicating with older persons.** A primary caregiver who is a student nurse mentioned that
266 he learned how to communicate effectively with older persons during the outreach component of
267 her nursing programme due to the emotional and practical care he provides for his grandmother.
268 *“When we go on outreach and those things, when I meet older persons, I communicate*
269 *with them in a way they feel like I’m their child and I believe all these things*
270 *[communication skills] are because of the interaction and care I provide to my*
271 *grandmother.”* (Male, 25 years, Primary caregiver, James Town)
272 272
273 **Enhanced personal attributes.** The primary and secondary caregivers were of the view that
274 caregiving to older persons enhanced their attributes. They expressed enhanced personal
275 attributes in four ways: (1) appreciation; (2) feeling valued; (3) humility and patience; and (4)
276 compassion.
277 **Appreciation.** Four family caregivers (3 primary and 1 secondary) explained that they felt
278 appreciated as relatives and non-relatives recognised their caregiving efforts that had improved
279 the general well-being of their care recipients. All the caregivers who felt appreciated were
280 females. A primary caregiver commented that:
281 *“People even tell me that I have done well by taking care good care of my mum. During*
282 *the Easter celebration, we went to church and when people saw her, they gave me a*
283 *handshake that I have done well by taking good care of my mum. When they do that, I*
284 *feel appreciated.”* (Female, 40 years, Primary caregiver, James Town)
285 **Feeling valued.** Family caregivers sometimes accompany their care recipients to family
286 meetings. In Ga Mashie and other parts of Ghana, attending and participating in family meetings
287 are often reserve for older persons. However, two female caregivers had the privilege to attend
288 family meetings with their care recipients and this made them feel valued.
289 *“… I feel I am important because I get to sit at places where I would not sit if I was*
290 *alone. I get to sit at the high table when I accompany her to family meetings. If it was not*
291 *because of her, I will not be sitting there because my age doesn’t qualify me.”* (Female,
292 53 years, Secondary caregiver, James Town)
293 *“Because of the care I provide to my care recipient, I can attend family meetings. If not*
294 *for her, my age would have disqualified me from attending such meetings.”* (Female, 39
295 years, Primary caregiver, Ussher Town)
296 **Humility and patience.** Three caregivers (2 primary and 1 secondary) stated that providing care
297 to older persons helped them to cultivate humility and patience. All these caregivers were
298 females. One secondary caregiver narrated that:
299 *“It has taught me to be humble and patient in whatever I do.”* (Female, 68 years,
300 Secondary caregiver, James Town)
301 **Compassion.** According to a female secondary caregiver, providing care to her father has helped
302 her to cultivate compassion towards the needy and assisting them when the means are available.
303 She reported that:
304 *“The help I give to him has helped me learn a lot of things. Some of the things that I have*
305 *learned are that if someone is in need and you are capable you should help him or her.”*
306 (Female, 23 years, Secondary caregiver, James Town)
307 307
308 **Family cohesion**. One female primary caregiver explained that before assuming her caregiving
309 roles and responsibilities, there used to be quarrels within her family, however, provision of care
310 to her mother has bonded the family.
311 *“There used to be some quarrels in the family but since I started providing care to my*
312 *mum it has brought the family together.”* (Female, 40 years, Primary caregiver, James
313 Town)
314 314
315 **Health consciousness.** The caregivers who provided health-related care often accompanied their
316 care recipients to hospitals/clinics for health checkups and this provided them the opportunity to
317 be educated on health conditions affecting their care recipients. One primary caregiver who cared
318 for her mother diagnosed with hypertension and diabetes explained that access to health
319 education on these conditions made her cautious of her diet and took the necessary precautionary
320 measures to reduce her risk of developing hypertension and diabetes. She noted:
321 *“Health-wise, I have learned a lot of things that affect the family that can equally affect*
322 *me so I am taking precautions now and then to make sure I don’t fall a victim. So I check*
323 *my diet.”* (Female, 44 years, Primary caregiver, Ussher Town)

# 324 Discussion

325 This study explored the positive experiences of primary and secondary caregivers in resource-limited
326 urban settings in Accra, the capital of Ghana. It sought to contribute to researchers’ and
327 policymakers’ understanding of the nuances of caregiving experiences and why caregivers
328 continue to provide care to older persons despite their negative experiences which are often
329 reported in the family caregiving literature. Although caregivers may experience some negative
330 outcomes from caregiving, they derive benefits (both tangible and intangible) which motivate
331 them to continue their challenging caregiving roles and responsibilities..
332 The findings from study showed that caregivers received gifts from their
333 care recipients, relatives, non-relatives, and religious institutions (such as the church) as a token
334 of appreciation for their caregiving roles and responsibilities. In the Ghanaian setting, the older
335 persons are often given food items, especially bread, when they are visited by their relatives and
336 non-relatives. Caregivers of older persons tend to benefit from these food items. In addition,
337 some older persons occasionally support their caregivers financially. This finding corroborates
338 previous studies in the United States which indicated that caregivers received financial support
339 from their care recipients [40, 43].
340 This study found that the family caregivers also received real estate as a reward for the care they
341 provide to their care recipients, namely: land and free room/accommodation. This finding is
342 significant because it is absent in the family caregiving literature, especially in Ghana and the
343 rest of sub-Saharan Africa.
344 The caregivers believed that the provision of care to older persons brought blessings to their
345 lives. This belief may be linked to their religiosity and/or spirituality. Previous studies in the
346 United States also found that caregivers perceived caregiving roles and responsibilities as a
347 blessing [41, 44].
348 In addition, the findings showed that caregiving to older persons provided family caregivers the
349 opportunity to learn skills such as performing household chores (for example, cooking and
350 cleaning), caring for older persons, and communicating with older persons. All the caregivers
351 who reported that they learned skills through caregiving had no prior caregiving experience. This
352 finding corroborates Fruhauf et al.’s [43] study in the United States which found that family
353 caregiving helped caregivers to acquire the requisite skills to provide care to their care recipients.
354 In Netto et al.’s [45] study in Singapore, family caregivers reported that providing care to older
355 persons enhanced their communication with other older persons.
356 Also, the study found that a caregiver became health conscious due to her caregiving roles and
357 responsibilities. Through her interaction with health professionals, she became knowledgeable
358 about hereditary conditions and consequently, became mindful of her diet and thereby reduced
359 her risk of developing those conditions. This finding corroborates Thornton and Hopp’s [18]
360 study in the United States which indicated that daughters became knowledgeable about their
361 parents' health conditions which could help prevent or reduce their risk of developing those
362 conditions.
363 The findings further showed that caregivers felt appreciated and valued while cultivating
364 humility and patience, and compassion towards the impoverished. The efforts of caregivers to
365 improve the general well-being of their care recipients were recognised by others and this made
366 the caregivers feel appreciated and pride in their caregiving roles. This result supports previous
367 studies in Hong Kong, Portugal, and the United States where family caregivers reported that they
368 appreciated the recognition of their caregiving efforts by their care recipients and others [18, 46-
369 48]. Also, previous studies in Hong Kong, Singapore, and the United States found that providing
370 care to care recipients helped family caregivers to develop patience or become more patient [43,

373	45, 47, 49, 50].

374 Family caregivers also reported that caregiving to their care recipients had strengthened their
375 family bond, especially among siblings. Since family members shared their caregiving roles and
376 responsibilities among themselves, they collaborate to enhance the well-being of their care
377 recipient and this strengthened the bond among them. This finding supports previous studies that
378 found that family caregiving enhanced the relationship among caregivers, care recipients, and
379 other relatives [18, 45, 50-53].
380 Concerning Kramer’s model of caregiver adaptation adopted for this study, the findings showed
381 that caregiver characteristics (such as age and gender) are linked to some positive outcomes
382 (gifts and skills acquisition). With respect to gifts, the findings show that there were some gender
383 dimensions. For example, only female caregivers received gifts in cash and in-kind while only
384 male caregivers received gifts in a form of land and access to room/accommodation. Male
385 caregivers often provide financial care [54-56] and this may explain why only female caregivers
386 received gifts in cash and in-kind. This finding suggests that interventions to promote positive
387 outcomes of caregiving should consider the gender dimensions of family caregiving and the
388 associated rewards.
389 Regarding age, all family caregivers who learned skills through family caregiving were young
390 and middle-aged adults (25 – 45 years). This suggests that the acquisition of skills during
391 caregiving may vary by age, and it is important that specific interventions to promote positive
392 outcomes of caregiving focus on the age of caregivers.

## 393 Limitation

394 This study has two main limitations. Firstly, the caregivers were purposively sampled and
395 consequently, the findings cannot be generalised to all resource-limited urban settings in Accra,
396 Ghana. However, the aim of this study is not to generalise findings but highlight the positive
397 experiences of caregivers in such a setting. Secondly, the cross-sectional nature of the study did
398 not allow the researchers to understand the complexities of family caregiving over a long period.
399 Despite these limitations, this study has provided comprehensive information on the positive
400 experiences of both primary and secondary caregivers and thus contributes to the literature on
401 this neglected aspect of caregiving in Ghana and the rest of sub-Saharan Africa.

## 402 Conclusions

403 This study demonstrated that there are positive experiences that people derive from providing care to older persons in the various communities. Family caregivers received gifts, blessings, acquired skills,
404 and became health conscious. In addition, caregiving enhanced family bonds, and personal traits
405 of the caregivers.
406 Family caregiving situation is complex and it alternates, hence future studies are needed to
407 explore the positive experiences of primary and secondary caregivers over time. Longitudinal
408 studies will help researchers and policymakers understand the complexities of positive
409 experiences of caregivers, and consequently, help design appropriate interventions to improve
410 the well-being of family caregivers and their care recipients.
411 412

# 412 Acknowledgements

413 The authors would like to thank all participants and research assistants for making this study
414 possible.

# 415 References

416 1.	United Nations Population Division. World population ageing 2019
417 (ST/ESA/SER.A/444). New York: United Nations; 2020.
418 2.	Population Reference Bureau. 2021 world population data sheet. Washington, DC:
419 Population Reference Bureau; 2021.
420 3.	Ghana Statistical Service (GSS). 2010 Population and housing census report: The elderly
421 in Ghana. Accra: Ghana Statistical Service; 2013.
422 4.	World Health Organization. World report on ageing and health. Geneva: World Health
423 Organization; 2015.
424 5.	van der Geest S. Will families in Ghana continue to care for older people? Logic and
425 contradiction in policy. In: Hoffman J, Pype K, editors. Ageing in sub-Saharan Africa: Spaces
426 and practices of care. London: Policy Press; 2016. p. 21-41.
427 6.	Gurayah T. Caregiving for people with dementia in a rural context in South Africa. South
428 Africa Family Practice. 2015;57(3):194–7.
429 7.	Feinberg L, Reinhard SC, Houser A, Choula R. Valuing the invaluable: The growing
430 contributions and costs of family caregiving (2011 update). Washington, D. C.: AARP Public
431 Policy Institute; 2011.
432 8.	Kusi G, Boamah Mensah AB, Boamah Mensah K, Dzomeku VM, Apiribu F, Duodu PA.
433 Caregiving motivations and experiences among family caregivers of patients living with
434 advanced breast cancer in Ghana. PloS one. 2020;15(3):e0229683.
435 9.	Agyemang-Duah W, Mensah CM, Peprah P, Arthur F, Addai B, Abalo EM. Informal
436 health care: Examining the role of women and challenges faced as caregivers in rural and urban
437 settings in Ghana. Journal of Public Health. 2019;27(3):321-7.
438 10.	Asuquo EF, Akpan-Idiok PA. The exceptional role of women as primary caregivers for
439 people living with HIV/AIDS in Nigeria, West Africa. In: Cascella M, Stones MJ, editors.
440 Suggestions for addressing clinical and non-clinical issues in palliative care. London:
441 IntechOpen; 2020.
442 11.	Atobrah D, Adomako Ampofo A. Expressions of masculinity and femininity in
443 husbands’ care of wives with cancer in Accra. African Studies Review. 2016;59(1):175-97.
444 12.	Aboderin I. Ageing in West Africa. In: Uhlenberg P, editor. International handbook of
445 population aging. 1. New York: Springer Science & Business Media; 2009. p. 253-76.
446 13.	Nortey ST, Aryeetey GC, Aikins M, Amendah D, Nonvignon J. Economic burden of
447 family caregiving for elderly population in Southern Ghana: the case of a peri-urban district.
448 International journal for equity in health. International Journal for Equity in Health.

450	2017;16(1):16.

451 14.	Oppong C. Familial roles and social transformations: Older men and women in sub-
452 Saharan Africa. Research on Aging. 2006;28(6):654–68.
453 15.	Ghana Statistical Service (GSS). Ghana living standards survey round 6 (GLSS 6):
454 Poverty profile in Ghana (2005-2013). Accra: Ghana Statistical Service; 2014.
455 16.	Population Reference Bureau. Family caregiving. Today's Research on Aging.

456	2016(33):1-9.

457	17.	Pristavec T. The burden and benefits of caregiving: A latent class analysis. The

458	Gerontologist. 2019;59(6):1078-91.

459	18.	Thornton N, Hopp FP. “So I Just Took Over”: African American daughters' caregiving

460	for parents with heart failure. Families in Society: The Journal of Contemporary Social Services.

461	2011;92(2):211-7.

462	19.	Schulz R, Eden J. Family caregiving for an aging America. Washington, DC: The

463	National Academies Press; 2016.

464	20.	Vellone E, Fida R, Cocchieri A, Sili A, Piras G, Alvaro R. Positive and negative impact

465	of caregiving to older adults: A structural equation model. Professioni Infermieristiche.

466	2011;64(4):237-48.

467	21.	Mayor MS, Ribeiro O, Paúl C. Satisfaction in dementia and stroke caregivers: a

468	comparative study. Revista Latino-Americana de Enfermagem. 2009;17(5):620-4.

469	22.	Sanuade OA, Boatemaa S. Caregiver profiles and determinants of caregiving burden in

470	Ghana. Public Health. 2015;129(7):941-7.

471	23.	Kyei-Arthur F, Codjoe SNA. “Caring for the elderly is very difficult”: Challenges and

472	coping strategies of caregivers in urban poor Accra, Ghana. Clinical Nursing Research.

473	2021;30(5):662-9.

474 24.	Cheng ST, Haley WE. Coping with cognitive impairment and family caregiving:
475 Introduction to the special section. Psychology and Aging. 2020;35(1):1-7.
476 25.	Jeong A, Shin D, Park JH, Park K. What we talk about when we talk about caregiving:
477 The distribution of roles in cancer patient caregiving in a family-oriented culture. Cancer
478 Research and Treatment. 2019;51(1):141-9.
479 26.	Adewuya AO, Owoeye OA, Erinfolami AR. Psychopathology and subjective burden
480 amongst primary caregivers of people with mental illness in South-Western Nigeria. Social
481 psychiatry and psychiatric epidemiology. 2011;46(12):1251-6.
482 27.	Akyar I, Dionne-Odom JN, Ozcan M, Bakitas MA. Needs assessment for Turkish family
483 caregivers of older persons with cancer: first-phase results of adapting an early palliative care
484 model. Journal of Palliative Medicine. 2019;22(9):1065-74.
485 28.	Bassah N, Ubenoh US, Palle JN. An exploratory study of the knowledge and practices of
486 family caregivers in the care of the elderly at home in the Buea Health District, Cameroon.
487 Journal of Gerontology & Geriatric Research. 2018;7(3).
488 29.	Nance DC, Rivero May MI, Flores Padilla L, Moreno Nava M, Deyta Pantoja AL. Faith,
489 Work, and reciprocity: Listening to Mexican men caregivers of elderly family members
490 American Journal of Men's Health. 2018;12(6):1985-93.
491 30.	Zhong Y, Wang J, Nicholas S. Social support and depressive symptoms among family
492 caregivers of older people with disabilities in four provinces of urban China: The mediating role
493 of caregiver burden. BMC Geriatrics. 2020;20(1):1-10.
494 31.	Epps F. The relationship between family obligation and religiosity on caregiving.

495	Geriatric Nursing. 2014;35(2):126-31.

496	32.	Kramer BJ. Gain in the caregiving experience: Where are we? What next? The

497	Gerontologist. 1997;37(2):218-32.

498 33.	Kyei-Arthur F. Family caregiving: Experiences of caregivers and their elderly care
499 recipeints in urban poor communities in Accra, Ghana [Unpublished doctoral thesis]: University
500 of Ghana; 2017.
501 34.	Jawahir S, Tan EH, Tan YRo, Noh SNM, Ab Rahim I. The impacts of caregiving
502 intensity on informal caregivers in Malaysia: findings from a national survey. BMC Health

503	Services Research. 2021;21(1):1-16.

504	35.	Etters L, Goodall D, Harrison BE. Caregiver burden among dementia patient caregivers:

505	a review of the literature. Journal of the American Academy of Nurse Practitioners.

506	2008;20(8):423-8.

507 36.	Connors MH, Seeher K, Teixeira‐ Pinto A, Woodward M, Ames D, Brodaty H.
508 Dementia and caregiver burden: a three‐ year longitudinal study. International Journal of
509 Geriatric Psychiatry. 2020;35(2):250-8.
510 37.	Dionisio KL, Arku RE, Hughes AF, Vallarino J, Carmichael H, Spengler JD, et al. Air
511 pollution in Accra neighborhoods: Spatial, socioeconomic, and temporal patterns. Environmental
512 Science and Technology. 2010;44(7):2270-6.
513 38.	CHF International and Accra Metropolitan Assembly (AMA). Accra poverty map: A
514 guide to urban poverty reduction in Accra. Accra: CHF International and Accra Metropolitan
515 Assembly (AMA); 2010.
516 39.	Attride-Stirling J. Thematic networks: an analytic tool for qualitative research.
517 Qualitative Research. 2001;1(3):385-405.
518 40.	Dellmann‐ Jenkins M, Blankemeyer M, Pinkard O. Young adult children and
519 grandchildren in primary caregiver roles to older relatives and their service needs. Family

520	Relations. 2000;49(2):177-86.

521	41.	Bennett S, Sheridan M, Richardson F. Caregiving as ministry: Perceptions of African

522	Americans providing care for elders. Families in Society: The Journal of Contemporary Social

523	Services. 2014;95(1):51-8.

524 42.	Morse JM. Critical analysis of strategies for determining rigor in qualitative inquiry.
525 Qualitative Health Research. 2015;25(9):1212-22.
526 43.	Fruhauf CA, Jarrott SE, Allen KR. Grandchildren’s perceptions of caring for
527 grandparents. Journal of Family Issues. 2006;27(7):887-911.
528 44.	Shim B, Barroso J, Gilliss CL, Davis LL. Finding meaning in caring for a spouse with
529 dementia. Applied Nursing Research. 2013;26(3):121-6.
530 45.	Netto NR, Jenny GYN, Philip YLK. Growing and gaining through caring for a loved one
531 with dementia. Dementia. 2009;8(2):245-61.
532 46.	Ribeiro O, Paul C. Older male carers and the positive aspects of care. Ageing & Society.

533	2008;28(2):165-83.

534 47.	Pang RC, Lee DT. Finding positives in caregiving: the unique experiences of Chinese
535 spousal caregivers of persons with young-onset dementia. Dementia. 2019;18(5):1615-28.
536 48.	Lopez–Anuarbe M, Kohli P. Understanding male caregivers’ emotional, financial, and
537 physical burden in the United States. Healthcare 2019;7(2):72.
538 49.	Sanders S. Is the glass half empty or half full? Reflections on strain and gain in
539 caregivers of individuals with Alzheimer's disease. Social Work in Health Care. 2005;40(3):57-

540	73.

541 50.	Cheng S-T, Mak EP, Lau RW, Ng NS, Lam LC. Voices of Alzheimer caregivers on
542 positive aspects of caregiving. The Gerontologist. 2016;56(3):451-60.
543 51.	Peacock S, Forbes D, Markle-Reid M, Hawranik P, Morgan D, Jansen L, et al. The
544 positive aspects of the caregiving journey with dementia: Using a strengths-based perspective to
545 reveal opportunities. Journal of Applied Gerontology. 2010;29(5):640-59.
546 52.	Johannessen A, Möller A. Experiences of persons with early-onset dementia in everyday
547 life: A qualitative study. Dementia. 2011;12(4):410-24.
548 53.	Wawrziczny E, Pasquier F, Ducharme F, Kergoat MJ, Antoine P. From ‘needing to
549 know’to ‘needing not to know more’: An Interpretative Phenomenological Analysis of couples'
550 experiences with early‐ onset Alzheimer's disease. Scandinavian Journal of Caring Sciences.

551	2016;30(4):695-703.

552	54.	van der Geest S. Respect and reciprocity: Care of elderly people in rural Ghana. Journal

553	of Cross-Cultural Gerontology. 2002;17:3-31.

554	55.	Wong OMH. Gender and intimate caregiving for the elderly in Hong Kong. Journal of

555	Aging Studies. 2005;19(3):375-91.

556	56.	Evans BC, Belyea MJ, Ume E. Mexican American males providing personal care for

557	their mothers. Hispanic Journal of Behavioral Sciences. 2011;33(2):234-60.

558

Figure	[Click here to access/download;Figure;Fig 1.docx](https://www.editorialmanager.com/pone/download.aspx?id=29821220&guid=02e57a3f-a4ae-4e50-be39-f22295ad685a&scheme=1)

Positive outcomes

Appraisal of role gain

##### Fig 1. Conceptual framework for positive experiences of primary and secondary caregivers

**Background and context**

**Intervening processes**

**Well-being outcomes**

Caregiver attitudinal variables and effort

Caregiver characteristics/other life responsibilities

Resources

Care receiver characteristics/ potential stressors

Supporting Information

[Click here to access/download](https://www.editorialmanager.com/pone/download.aspx?id=29841033&guid=88ecaab3-465d-4622-9a30-2fff6a7e1bb8&scheme=1)

[**Supporting Information** S1 Interview Guide.docx](https://www.editorialmanager.com/pone/download.aspx?id=29841033&guid=88ecaab3-465d-4622-9a30-2fff6a7e1bb8&scheme=1)

Supporting Information

#### [Click here to access/download](https://www.editorialmanager.com/pone/download.aspx?id=29841042&guid=43e5bf8c-1df7-4a75-ae14-042814927632&scheme=1)

[**Supporting Information** S2 Appendix.docx](https://www.editorialmanager.com/pone/download.aspx?id=29841042&guid=43e5bf8c-1df7-4a75-ae14-042814927632&scheme=1)
